# Lord Dawson on Hospital Problems

**Published:** 1924-08

**Authors:** 


### LORD DAWSON ON THE MODEL HOSPITAL.

On July 16 Lord Dawson of Penn addressed a large number of West of England doctors who were paying a visit to the Forbes Fraser Hospital, Combe Park, Bath. This was formerly known as the Royal United Hospital and was visited recently by the Duke of Connaught. Lord Dawson spoke of the attributes which gave Combe Park its distinction, and spoke first of the adequate ground accommodation. If there was one condition which needed to be driven home more than another, it was that any hospital of to-day, especially a newly constructed hospital, should be erected outside a town, away from the hustle and din of life. A further attribute of a building like that, which its founders had been wise enough to think about, was cost. Those simple buildings answered their purpose well.

By adopting that plan of construction, they got a hospital at something like half the cost of the flexible hospitals to be found in the bigger towns. It was probably less than that. The cost of that hospital worked out upon construction about £500 a bed, and they would not get a hospital on the many-storeyed system under from £1,000 to £1,200 per bed. There was the further advantage that it could be scrapped, modified or altered quite easily, whereas the hospital which was constructed on the older lines had to stand for at least a generation before anybody felt justified in interfering with it. So not only did we get cheaper construction, but greater flexibility. Turning to another aspect, which he termed the more professional aspect of the hospital problem, Lord Dawson said that scheme had among its aims the bringing of the doctors of Bath and district into closer contact with hospital life. They had passed from the realm of individual labour into the realm of team work, which meant they must have fabric and organisation to that end for their different departments, side by side. There was no doubt in his mind that illnesses would in future be much more often treated in institutions like that than they would be in the homes of patients.

